# Platelet‐rich fibrin matrix as treatment for complex anal fistulae: A 3‐year experience at a tertiary centre

**DOI:** 10.1111/codi.70447

**Published:** 2026-04-03

**Authors:** Alejandro Lusilla Lopez, Ursula Aho Fält, Pamela Buchwald, Louis Banka Johnson, Olof Grip

**Affiliations:** ^1^ Department of Surgery Skåne University Hospital Malmö Sweden; ^2^ Department of Clinical Sciences, Malmö Lund University Malmö Sweden; ^3^ Department of Gastroenterology Skåne University Hospital Malmö Sweden

**Keywords:** complex anal fistula, Crohn's disease, regenerative fistula treatment

## Abstract

**Aim:**

Treatment of complex anal fistulae remains a significant challenge for physicians and patients. A sphincter‐preserving technique with bioactive platelet‐rich fibrin matrix is one option. This three‐year study aimed to evaluate the efficacy of bioactive platelet‐rich fibrin matrix in treating complex anal fistulae.

**Method:**

This retrospective study analysed data from 2019 to 2022 of patients diagnosed with complex anal fistulae treated with a platelet‐rich fibrin matrix at a tertiary centre. Endoanal ultrasound was used to morphologically characterize fistulas using Park's classification. Pearson's chi‐squared and Kaplan–Meier (log‐rank) analyses were used to evaluate between‐group differences.

**Results:**

A total of 213 patients were included in the study (181 with cryptoglandular fistulae and 32 with Crohn's disease‐associated fistulae) with a median follow‐up of 6.6 months (range 0.5–67.1 months). Cavities and secondary tracts were identified in 14.6% and 34.7% of the patients, respectively. External opening closure occurred in 32% after one procedure and 43% cumulatively after a second intervention. Healing rates did not differ statistically by aetiology or fistula location. Cavities and secondary tracts were associated with failure. During follow‐up, 23% of the secondary tracts healed, whereas cavities derived no significant benefit from the intervention. No serious complications were reported.

**Conclusion:**

Platelet‐rich fibrin matrix is a simple and safe sphincter‐preserving technique that achieves reasonably good results in the treatment of cryptoglandular and Crohn's disease‐associated fistulae. While secondary tracts may respond well, further research is required to optimize the management of fistulae associated with cavities.

**What does this paper add to the literature?**

This is the largest reported cohort to date of patients treated with bioactive platelet‐rich fibrin matrix and indicates that the method is safe, simple and provides reasonably good results for treating both cryptoglandular and Crohn's‐related fistulae.

## INTRODUCTION

An anal fistula is a pathological tract between the anal canal and the perianal skin. Cryptoglandular fistulae originate in the intersphincteric glands according to the cryptoglandular theory [1]. The majority of these initially present as anal abscesses; however, only 16% manifest as a fistula [2]. The likelihood of developing a fistula in patients with Crohn's disease is approximately twice as high [2]. Anal fistulae have been classified according to Park's classification to establish the type and proportion of the sphincters involved [3]. This classification, in combination with clinical and imaging assessment, is useful for selecting the appropriate treatment method. Fistulotomy represents the most effective method, with a healing rate of approximately 94% [4].

However, fistulotomy may result in high incontinence rates in patients with fistulae involving a substantial portion of the anal sphincters [5]. To preserve continence, numerous sphincter‐sparing procedures such as advancement flap, ligation of the intersphincteric fistula tract (LIFT), or laser techniques have been developed [6].

Biological treatments including fistula plug, platelet‐rich plasma and autologous or allogenic stem cell transplantation have also gained prominence for complex fistulae [7, 8, 9]. One such biological treatment is Obsidian® Regenerative Fistula Treatment (RFT), an autologous platelet‐rich fibrin (PRF) matrix. This structure is achieved using specific polymerization agents (tranexamic acid and Batroxobin), which allow for a prolonged release of growth factors (5–7 days) from non‐activated platelets. This sustained bioactive profile is designed to support regeneration over a longer window than standard autologous preparations [10]. Obsidian® RFT has been applied in various surgical procedures, including hepatectomy, inguinal hernia repair and colorectal surgery, demonstrating efficacy in healing and reducing complications [11, 12, 13]. While other autologous compound PRF foams have been studied in patients with complex fistulae, only two published studies report on Obsidian® RFT, both with limited patient numbers [14, 15].

This study aimed to analyse the efficacy and safety of PRF matrix with Obsidian® RFT in a large cohort of patients with complex fistulae.

## METHODS

This retrospective study was conducted at a tertiary referral centre, between 2019 and 2022. Although Obsidian RFT® remains in routine clinical use at our institution, study inclusion was restricted to procedures performed through the end of 2022. This cutoff was established strictly to ensure a robust long‐term follow‐up period for the entire cohort.

The study was conducted and reported in accordance with the Strengthening the Reporting of Observational Studies in Epidemiology (STROBE) statement [16]. Patients were diagnosed at the outpatient clinic, and all underwent examination with three‐dimensional (3D) endoanal ultrasound (EAUS). The anatomy of the fistula was categorized according to the Park's classification.

PRF matrix was offered to patients with middle‐high transphincteric, suprasphincteric, extrasphincteric fistulae or intersphincteric fistulae in patients with some incontinence symptoms that refused fistulotomy. Fistulae associated with cavities or secondary tracts were also considered for PRF matrix after adequate drainage. Only highly symptomatic or inflamed fistulae, or those presenting with sepsis, were drained with a loose seton prior to definitive surgery. PRF matrix was also offered to patients with Crohn's disease‐related fistulae. These patients received biological therapy by gastroenterologists prior to surgery and underwent treatment with PRF matrix only once inflammation was adequately controlled after clinical and on 3D EAUS examination.

Postoperatively, all patients were scheduled for routine follow‐up including clinical and 3D EAUS examination.

Patients under 18 years of age and those affected by fistulae related to other aetiology were excluded.

Fistula healing, as primary outcome, was defined as complete closure of the external opening without any signs of fluid discharge. Secondary endpoints included adverse events related to the treatment and the identification of factors associated with the absence of healing, such as aetiology, fistula location and the presence of secondary tracts or cavities. Radiological healing was additionally assessed by EAUS and defined as the replacement of the fistulous tract with homogeneous tissue of intermediate echogenicity, in accordance with the criteria proposed by Cricrì et al. [17].

Data were collected from a secured electronic patient filing system. The study was approved by the Swedish Ethical Review Authority; Stockholm Dnr 2024‐03797‐01.

### PRF matrix preparation and surgical technique

On the day of surgery, 120 mL of blood was taken from patients using a designated preparation chamber. Subsequently, the matrix was prepared according to the manufacturer's instructions [10]. The surgical procedure involved debriding and cleaning of the fistula tract. The internal opening was sutured using absorbable sutures, and a leakage test was performed to ensure complete closure of the internal opening before placing the matrix. The matrix was then sprayed into the fistula tract using a special applicator connected to the machine housing the platelet solution. The final solution had a gluey, fibrin‐like consistency. The internal fistula opening was closed with the previously placed sutures. The external fistula opening was excised and the wound left open.

### Statistical analysis

No formal sample size calculation was conducted. Instead, all consecutive patients meeting the inclusion criteria during the study period were included. Comparisons between categorical variables were conducted using Pearson's chi‐squared test. Kaplan–Meier analysis with log‐rank test was employed to assess potential differences in fistula recurrence between cryptoglandular and Crohn's‐related fistulae. Statistical significance was defined as *p* < 0.05. All analyses were performed using IBM SPSS software (version 28.0.1.1). Kaplan–Meier curve was generated to illustrate fistula healing throughout the follow‐up period.

## RESULTS

The study included a total of 214 patients, comprising 181 patients with cryptoglandular fistulae and 33 with perianal Crohn's disease. One patient declined participation in the study and was excluded.

Demographics, patient and fistula characteristics at baseline are provided in Table 1. Specific disease phenotypes, including the Montreal classification and medical management for the Crohn's disease subgroup, are detailed in Table S1.

**TABLE 1:** Baseline patient demographics and clinical characteristics.

	Crohn's	Cryptoglandular	Total
*N* (%)	32 (15.0)	181 (85.0)	213 (100.0)
Age (SD)	41 (14)	46 (13)	45 (14)
Gender			
Female	16 (50.0)	66 (36.5)	82 (38.5)
Male	16 (50.0)	115 (63.5)	131 (61.5)
Diabetes			
No	32 (100.0)	166 (91.7)	198 (93.0)
Yes	0 (0.0)	15 (8.3)	15 (7.0)
Smoking			
No	22 (68.8)	143 (79.0)	165 (77.5)
Yes	10 (31.2)	38 (21.0)	48 (22.5)
Anti‐TNF alpha treatment			
No	2 (6.2)	176 (97.2)	178 (83.6)
Yes	30 (93.8)	5 (2.8)	35 (16.4)
Faecal diversion			
No	28 (87.5)	175 (96.7)	203 (95.3)
Yes	4 (12.5)	6 (3.3)	10 (4.7)
Type of fistula			
Intersphicteric	0 (0.0)	1 (0.6)	1 (0.5)
Transphincteric	31 (96.9)	174 (96.1)	205 (96.2)
Suprasphincteric	1 (3.1)	4 (2.2)	5 (2.3)
Extrasphincteric	0 (0.0)	2 (1.1)	2 (0.9)
Orientation			
Dorsal	18 (56.2)	93 (51.4)	111 (52.1)
Ventral	7 (21.9)	73 (40.3)	80 (37.6)
Left lateral	6 (18.8)	8 (4.4)	14 (6.6)
Right lateral	1 (3.1)	7 (3.9)	8 (3.8)
Horseshoe fistula			
No	29 (90.6)	173 (95.6)	202 (94.8)
Yes	3 (9.4)	8 (4.4)	11 (5.2)
Cavity			
No	25 (78.1)	157 (86.7)	182 (85.4)
Yes	7 (21.9)	24 (13.3)	31 (14.6)
Secondary tract			
No	18 (56.2)	121 (66.9)	139 (65.3)
Yes	14 (43.8)	60 (33.1)	74 (34.7)
Previous surgery			
No	22 (68.8)	115 (63.5)	137 (65.3)
Yes	10 (31.2)	66 (36.5)	76 (34.7)
Previous type of surgery			
None	23 (71.9)	116 (64.1)	139 (65.3)
Fistulotomy	0 (0.0)	2 (1.1)	2 (0.9)
Plug	5 (15.6)	17 (9.4)	22 (10.3)
Advancement flap	0 (0.0)	4 (2.2)	4 (1.9)
Several	4 (12.5)	42 (23.2)	46 (21.6)

*Note*: For categorical variables, data are presented as *n* (percentage).

A total of 309 surgical procedures with PRF matrix were performed with a median follow‐up of 6.6 months (range 0.5–67.1 months). Seventy‐three patients underwent a total of 2 procedures, 18 patients underwent 3 procedures, 4 patients underwent 4 procedures and 1 patient underwent 5 procedures. Six patients failed to return for scheduled follow‐up visits, precluding the assessment of healing. Overall healing rate after a single procedure was 32%, increasing to 43% following a second intervention and 47% following three to five operations. Among these patients with healed fistulae, median follow‐up was 45.3 months (range 4.0–67.1).

**FIGURE 1:**
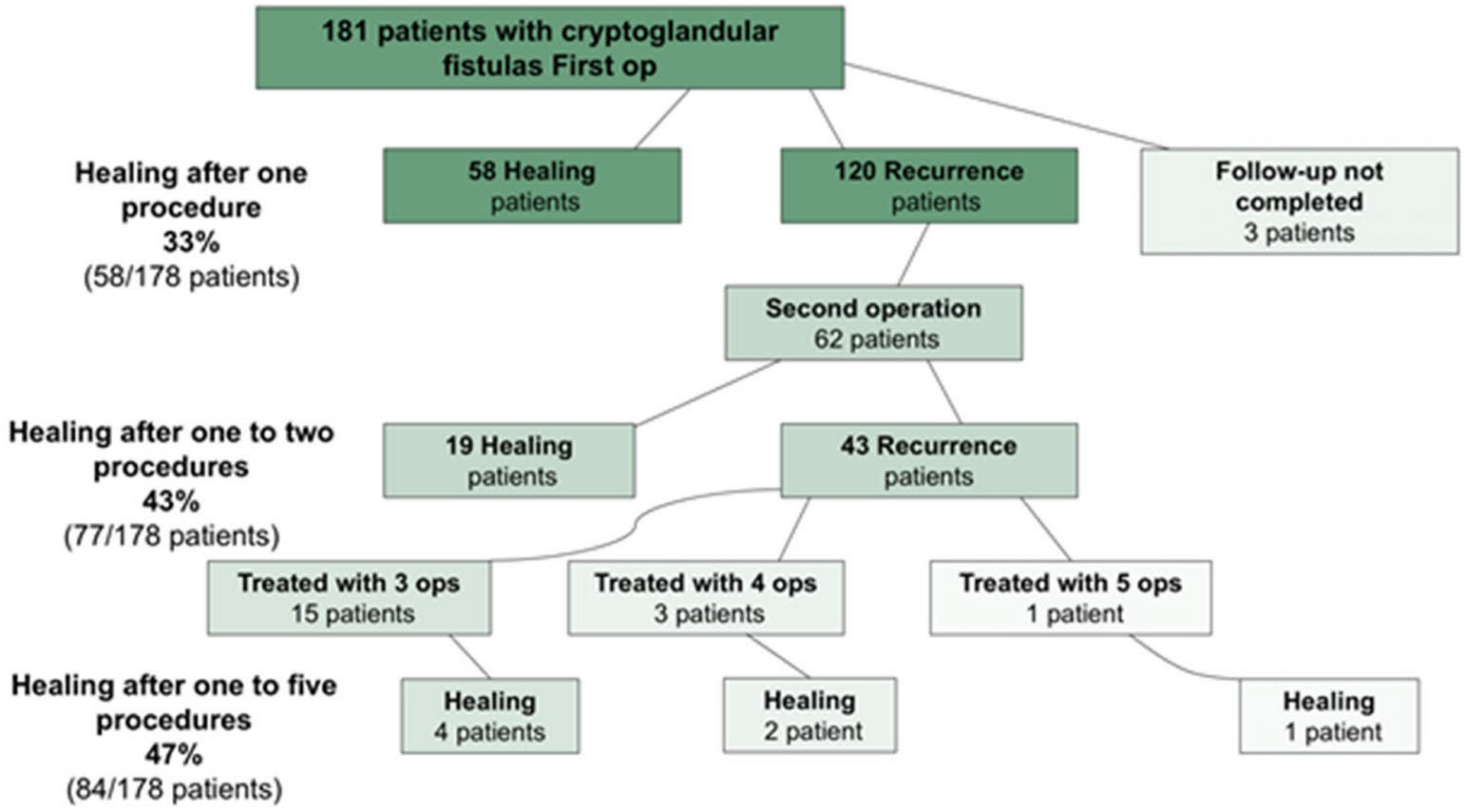
Treatment and result algorithm for patients with cryptoglandular fistulae.

Among patients with cryptoglandular fistulae, the overall healing rate after a single procedure was 33%, increasing to 43% following a second procedure and 47% following up to five procedures (Figure 1).

**FIGURE 2:**
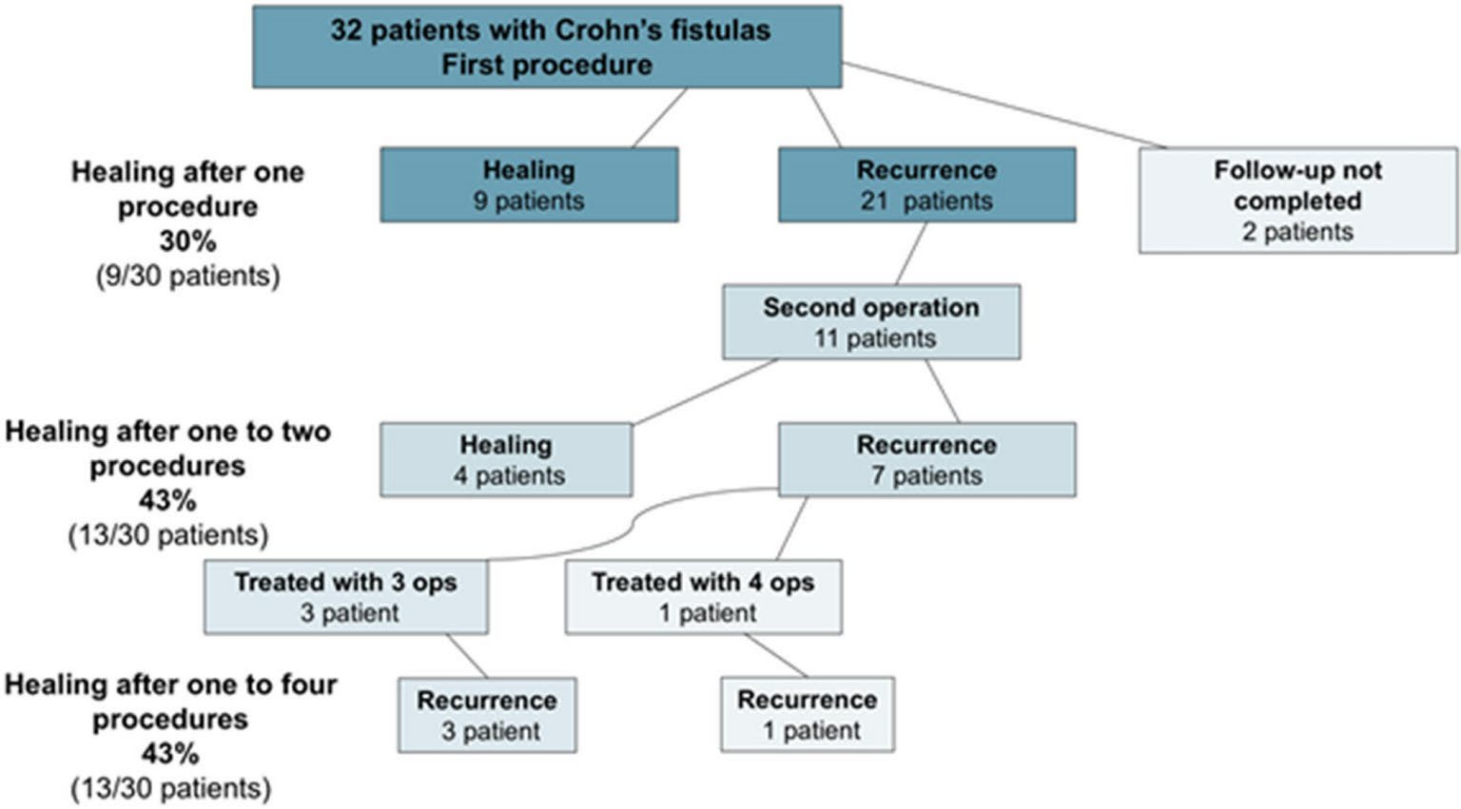
Treatment and result algorithm for patients with known Crohn's disease.

Among patients with Crohn's disease‐related fistulae, overall healing rate after a single procedure was 30%, increasing to 43% following a second procedure and 43% following four procedures (Figure 2).

Endoanal ultrasound (EAUS) confirmed anatomical closure in 74.8% of clinically healed patients. However, 23.2% of this subgroup exhibited a persistent hypoechoic tract despite the absence of clinical symptoms or an external opening. The remaining 2.0% were excluded from radiological analysis as they declined the postoperative ultrasound examination.

There were no reported cases of severe systemic complications, sepsis, or mortality within the 30‐day postoperative period. Regarding local morbidity, 13 patients (6.3%) developed a perianal abscess requiring surgical drainage within 30 days of the procedure. When calculated per operative event, this represents an overall abscess formation rate of 4.2%. No other adverse events were recorded.

**FIGURE 3:**
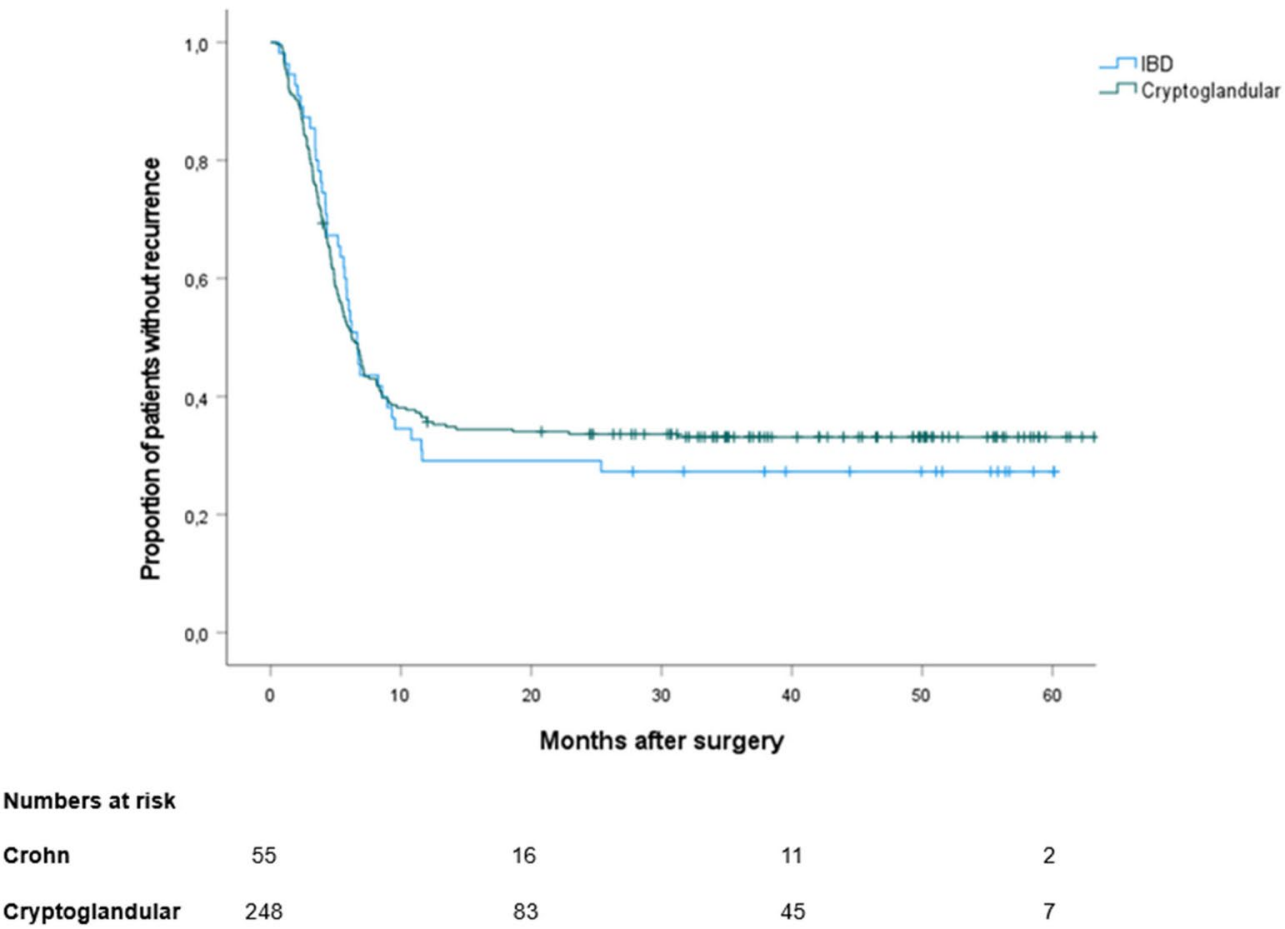
Fistula healing during follow‐up by aetiology. Kaplan–Meier curves show the cumulative probability of fistula healing over time, stratified by fistula aetiology and by the number of procedures performed. Tick marks denote censored observations; numbers at risk are displayed beneath the *x*‐axis.

Healing rates did not differ between cryptoglandular and Crohn's disease‐related fistulae (*p* = 0.378), as shown in the Kaplan–Meier curve during follow‐up (Figure 3). Fistula location was likewise not associated with healing (*p* = 0.187).

**FIGURE 4:**
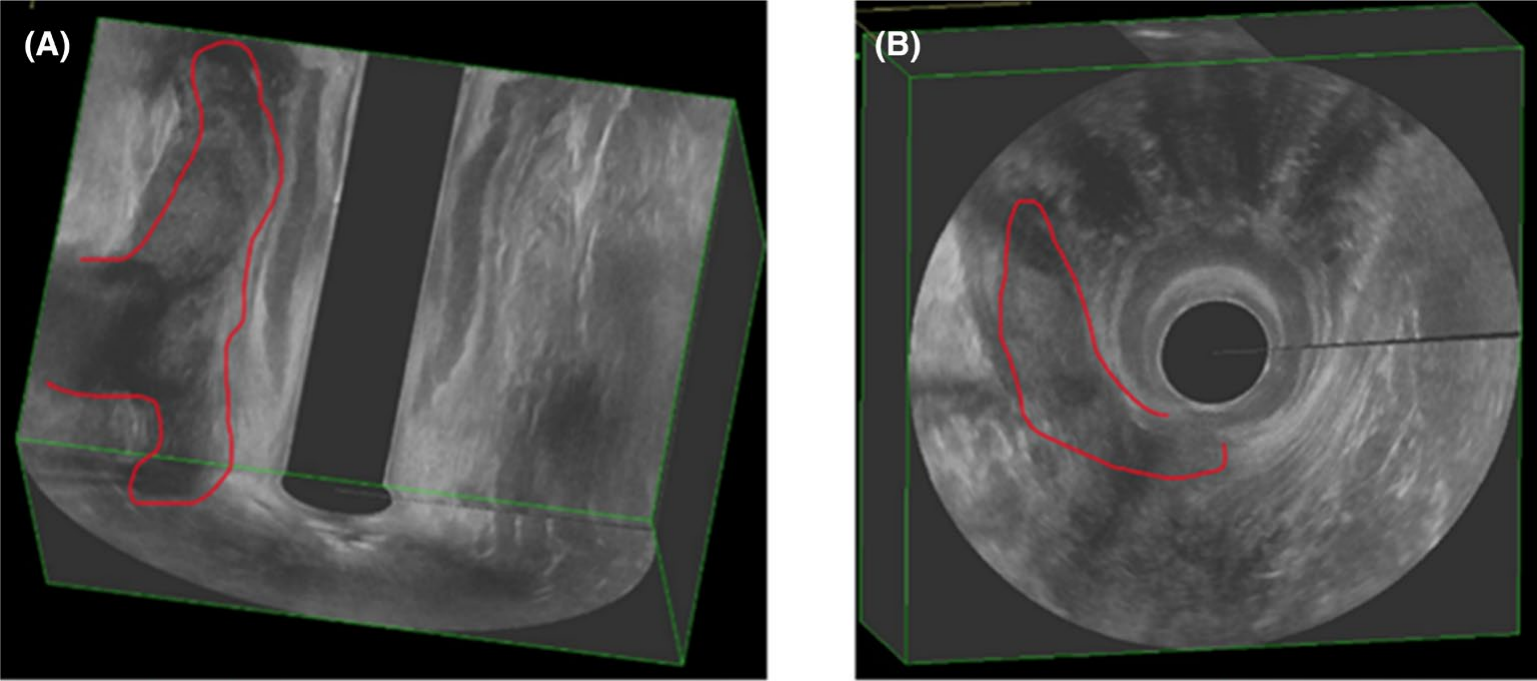
Endoanal ultrasound of a high transphincteric fistula with a large secondary tract. Three‐dimensional endoanal ultrasound. (A) Coronal plane showing the secondary tract along the anal canal (red contour). (B) Axial (transverse) slice of the anal canal with the corresponding tract outlined in red. The central artefact corresponds to the probe footprint.

**FIGURE 5:**
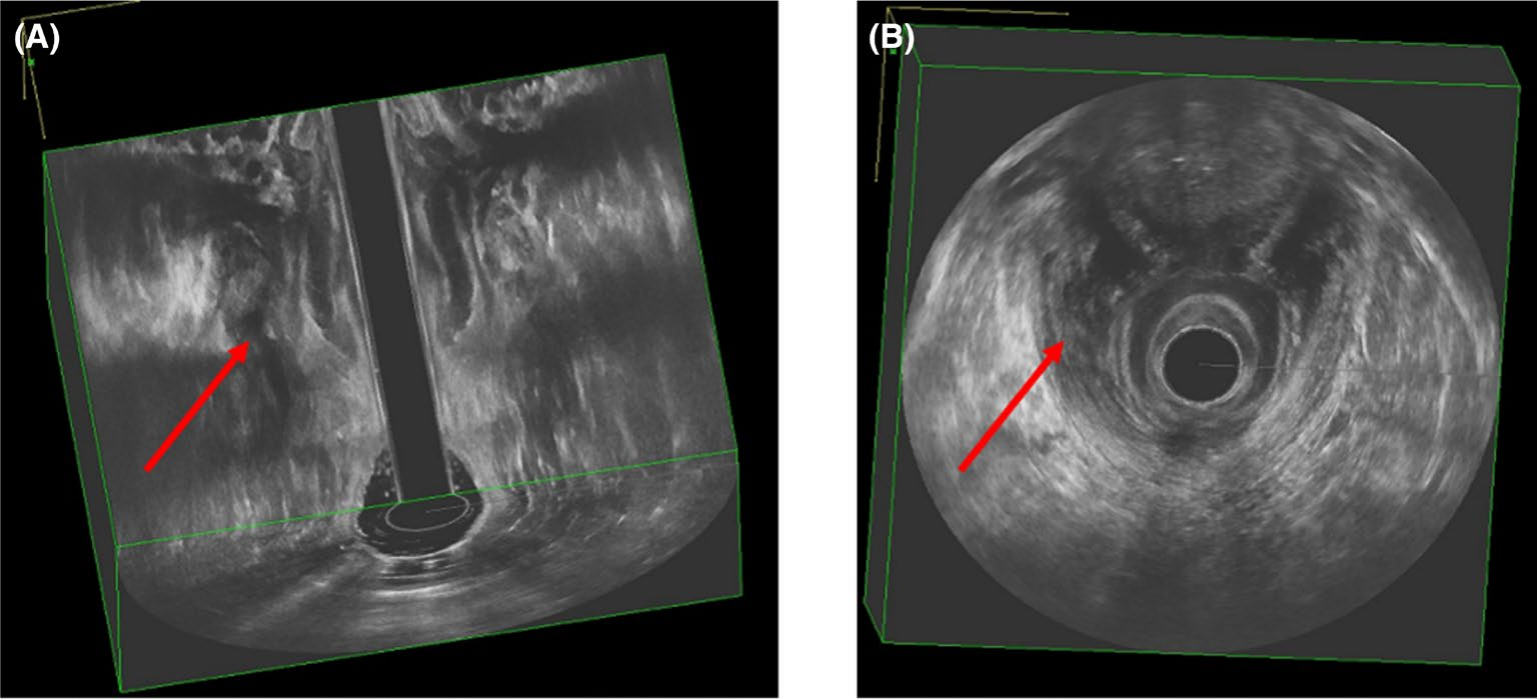
Endoanal ultrasound demonstrating closure of the secondary tract shown in Figure 4. (A) Coronal plane. (B) Axial (transverse) slice. Red arrows indicate the site of the previously outlined secondary tract; interval imaging shows no discrete tract. The central artefact corresponds to the probe footprint.

Cavities and secondary tracts were predictive of recurrence (*p* = 0.015 and *p* = 0.009 respectively). Although no significant improvement in cavities was observed (*p* > 0.05), 23% of fistulae with secondary tracts benefited from treatment through reduction or closure (*p* < 0.01, Figures 4 and 5).

## DISCUSSION

This study shows that, despite the complexity and heterogeneity of anal fistulae, treatment with PRF matrix yields encouraging outcomes in complex cases. To our knowledge, our cohort is one of the largest reported to date evaluating PRF‐based approaches in anal fistulae [18, 19, 20, 21, 22].

We additionally analysed outcomes in fistulae with cavities and/or secondary tracts, a subgroup seldom described in detail. Outside PRF‐based strategies, delta video‐assisted anal fistula treatment (dVAAFT) has reported promising healing in a small pilot by La Torre et al., with supportive commentary by Tozer [23]. In our cohort, cavities did not appear amenable to closure with PRF matrix, whereas secondary tracts could be obliterated or reduced, potentially downstaging overall fistula complexity.

Outcomes with PRF were similar in Crohn's disease–related and cryptoglandular fistulae. These comparable results likely reflect close collaboration with gastroenterologists to ensure adequate drainage and minimal inflammation, key principles in the management of perianal Crohn's disease before definitive intervention [24, 25]. By contrast, the meta‐analysis by Stellingwerf et al. reported lower success for Crohn's disease–related fistulae when treated with LIFT or endoanal advancement flap [25]. Our findings therefore suggest that PRF matrix is a safe and reasonably effective option in selected Crohn's‐related fistulae.

In our experience, a follow‐up of more than 6 months is important to reliably confirm healing. Several patients who appeared unhealed at the six‐month visit were subsequently found to be healed at examination under anaesthesia a few months later, underscoring the value of an initial wait‐and‐see approach and avoidance of unnecessary re‐intervention.

A notable observation was discordance between some 3D endoanal ultrasound (EAUS) findings and clinical status during follow‐up. In patients with closure of the external opening and no discharge or symptoms, we sometimes observed the persistence of a hypoechoic area at the site of the previous tract, whereas in other cases no tract was identifiable. For this study, we defined healing as external opening closure without discharge, irrespective of residual hypoechoic signal on 3D‐EAUS. One explanation for the persistence of a hypoechoic tract is that radiological resolution takes significantly longer than clinical closure. Consequently, a follow‐up period of less than 12 months may be insufficient to demonstrate complete radiological healing. Current literature suggests that MRI assessment is most reliable at 12–18 months, particularly in Crohn's disease‐associated fistulae [26, 27]. In addition, Iqbal et al. previously reported that MRI‐defined radiological healing is associated with long‐term outcomes [28], the predictive value of 3D‐EAUS findings described here warrants further evaluation. Given cost and capacity constraints around MRI, clarifying the role of EAUS in predicting recurrence is a priority for future research.

This study has limitations. First, its retrospective design combined with the absence of a control group (e.g., LIFT, advancement flap, laser ablation of fistula tract) limits causal inference and precludes direct effectiveness comparisons and adjustment for confounding. Second, heterogeneity, including fistulae with cavities and secondary tracts, may have reduced the observed healing rate. Achieving complete homogeneity is challenging given the nature of this condition. Although inclusion of Crohn's disease–related fistulae introduce additional heterogeneity, healing rates were similar between aetiologies, supporting PRF matrix as an additional therapeutic option for the complex clinical challenge of perianal Crohn's disease. Third, missing data and incomplete follow‐up (including variability in the timing and modality of assessment) may have led to imprecision or misclassification of healing outcomes. Fourth, selection bias is possible due to referral patterns and treatment selection in a tertiary setting, which may limit generalisability. Fifth, while EAUS was performed for routine assessment, the absence of MRI limits direct comparison with studies using MRI imaging. Sixth, Quality of Life (QoL) questionnaires were not collected at the time of follow‐up. Upcoming studies should include them in order to assess QoL variations after this treatment. Seventh, clinical disease activity indices (such as CDAI or PDAI) were not routinely recorded at the time of surgery for patients with Crohn's‐related fistulae. Future prospective studies should incorporate these scores to better characterize baseline disease severity and provide a more comprehensive clinical profile of this specific subgroup.

Bioactive PRF matrix is not currently included in the European cryptoglandular fistula guidelines treatment algorithm [29]. Considering our results, the absence of serious complications, and the procedural reproducibility, PRF matrix may be considered as an alternative to laser ablation of the fistula tract (LAFT) in cases where LAFT is unavailable or unsuitable (e.g., wide tracts or secondary tracts). Comparative studies are lacking, but PRF and LAFT may be complementary, offering sphincter‐sparing alternatives to LIFT or advancement flap.

In conclusion, bioactive PRF matrix with Obsidian® RFT is a safe, sphincter‐sparing method with overall favourable outcomes in complex anal fistulae. It may be considered as an alternative to LAFT in selected cases. Careful patient selection could further improve success rates. Well‐designed prospective cohort studies with strict inclusion criteria are needed to confirm efficacy and to determine its capacity to downstage complex fistulae with secondary tracts.

## AUTHOR CONTRIBUTIONS

**Louis Banka Johnson:** Conceptualization; methodology; writing – review and editing. **Ursula Aho Fält:** Methodology; data curation; formal analysis; visualization; writing – review and editing. **Alejandro Lusilla Lopez:** Conceptualization; methodology; formal analysis; investigation; data curation; visualization; writing – original draft. **Pamela Buchwald:** Methodology, writing – review and editing, supervision, funding acquisition. **Olof Grip:** Methodology, writing – review and editing, supervision, funding acquisition.

## FUNDING INFORMATION

The author Alejandro Lusilla Lopez is a PhD student at Lund University, Faculty of medicine (Sweden). This work was performed within the scope of the author's doctoral studies. This study was funded by regional research funds from Region Skåne.

## CONFLICT OF INTEREST STATEMENT

Alejandro Lusilla Lopez, Ursula Aho Fält, Pamela Buchwald, Louis Banka Johnson declares no conflict of interest. Olof Grip has received consulting fees from Abbvie, Bristol Mayers Squibb, Ferring, Johnson&Johnson, Pharmacosmos, Pfizer, Tillotts, Takeda.

## ETHICS STATEMENT

The study was approved by the Swedish Ethics Committee; Stockholm Dnr 2024‐03797‐01.

## PATIENT CONSENT STATEMENT

Oral/written informed consent was obtained from all individual participants included in the study.

## Supporting information


Table S1.

Data S1.

## Data Availability

The data that support the findings of this study are available from the corresponding author upon reasonable request.
