# Supplementary material for: Platelet‐rich fibrin matrix as treatment for complex anal fistulae: A 3‐year experience at a tertiary centre
Source: Colorectal Dis. 2026 Apr 3;28(4):e70447. doi: 10.1111/codi.70447 (PMC13048851; doi:10.1111/codi.70447)
Supplement: Supplementary file 1 — Table S1. [file CODI-28-0-s001.docx]

**Supplementary Table 1:** Baseline disease phenotype, defined by the Montreal classification, and medical therapy of the Crohn’s disease cohort.

A1; age <16 years; A2; age 17–40 years; A3; age >40 years.

Location of Crohn’s disease: L1, ileal; L2, colonic; L3, ileo-colonic; L4, upper GI tract disease.

Behaviour of Crohn’s disease: B1, non-stricturing; non-penetrating; B2, stricturing; B3, penetrating; p, perianal.

For categorical variables, data are presented as n (percentage).
